# Molecular detection and genetic diversity of *Leucocytozoon sabrazesi* in chickens in Thailand

**DOI:** 10.1038/s41598-021-96241-7

**Published:** 2021-08-17

**Authors:** Pacharaporn Khumpim, Runglawan Chawengkirttikul, Witchuta Junsiri, Amaya Watthanadirek, Napassorn Poolsawat, Sutthida Minsakorn, Nitipon Srionrod, Panat Anuracpreeda

**Affiliations:** 1 Parasitology Research Laboratory (PRL), Institute of Molecular Biosciences, Mahidol University, Nakhon Pathom, 73170 Thailand; 2 Department of Microbiology, Faculty of Science, Mahidol University, Bangkok, 10400 Thailand

**Keywords:** Parasitology, Environmental sciences

## Abstract

*Leucocytozoon sabrazesi* is the intracellular protozoa of leucocytozoonosis, which is transmitted by the insect vectors and affects chickens in most subtropical and tropical regions of the globe, except South America, and causing enormous economic losses due to decreasing meat yield and egg production. In this study, *L. sabrazesi* gametocytes have been observed in the blood smears, and molecular methods have been used to analyse the occurrence and genetic diversity of *L. sabrazesi* in blood samples from 313 chickens raised in northern, western and southern parts of Thailand. The nested polymerase chain reaction (nested PCR) assay based on the *cytb* gene revealed that 80.51% (252/313) chickens were positive of *L. sabrazesi*. The phylogenetic analysis indicated that *L. sabrazesi cytb* gene is conserved in Thailand, showed 2 clades and 2 subclades with similarity ranged from 89.5 to 100%. The diversity analysis showed 13 and 18 haplotypes of the sequences from Thailand and from other countries, respectively. The entropy analyses of nucleic acid sequences showed 26 high entropy peaks with values ranging from 0.24493 to 1.21056, while those of amino acid sequences exhibited 5 high entropy peaks with values ranging from 0.39267 to 0.97012. The results; therefore, indicate a high molecular occurrence of *L. sabrazesi* in chicken blood samples with the associated factors that is statistically significant (*p* < 0.05). Hence, our results could be used to improve the immunodiagnostic methods and to find appropriate preventive control strategies or vaccination programs against leucocytozoonosis in order to mitigate or eliminate the harmful impact of this infection on chicken industry.

## Introduction

Leucocytozoon is a haemosporidian protozoan that belongs to the phylum of Apicomplexa causing leucocytozoonosis in birds, including chickens. This parasite is found in blood cells of avian hosts, and transmitted by simuliid blackflies (Diptera: Simuliidae) or Culicoides midges (Diptera: *Ceratopogonidae*)^1–3^. The high pathogenic cases caused by *L. sabrazesi* exhibit chicken clinical mortality and subclinical decreased egg production with several symptoms including depression, weakness, anorexia, restlessness, anemia, pale comb, green feces and dead^4–10^. The infected chickens possess mortality rate more than 50% resulting in significant economic losses^9^. Outbreaks of *Leucocytozoon* infection have been reported in chicken (Asia and Africa), waterfowl (Asia, Europe and North America), turkeys (North America), and free-living and captive avian species throughout the globe^7,11–16^. In Thailand, the first case of leucocytozoonosis was reported by Campbell^17^ known as ‘Bangkok hemorhagic disease’ in chicken. There have been a few reports on *Leucocytozoon* sp. infections in both domestic chickens (42.86%) and wild birds (16.67–22.22%) in Chiang Mai and Phetchabun provinces, respectively^18,19^.

Conventional diagnostic method for *Leucocytozoon* sp. infection is direct microscopic observation of the circulating gametocytes in Giemsa-stained blood smear. In addition, detection of parasites DNA or RNA using polymerase chain reaction (PCR) method utilizing primers derived from parasites mitochondrial cytochrome b (*cytb*) gene could be more common reliable and widely used in diagnosing the infections particular in laboratory for high sensitivity and specificity even when blood smears are negative in case of low parasitemia or early stage of infection in animals^9,12,20–22^. Little is known about the prevalence of *Leucocytozoon sabrazesi* in Thailand. The aim of this study was to further investigate the occurrence and genetic diversity of this parasite in chickens from three regions (northern, western and southern) of Thailand. Likewise, haplotype diversity and entropy analysis among the isolated sequences discriminated in this study and those from other countries are investigated. These results could provide the information on the genetic diversity structure of this parasite’s population for further development of the immunodiagnostic methods and vaccine strategies.

## Results

### Occurrence of *L. sabrazesi* infection in chicken blood samples

**Figure 1:**
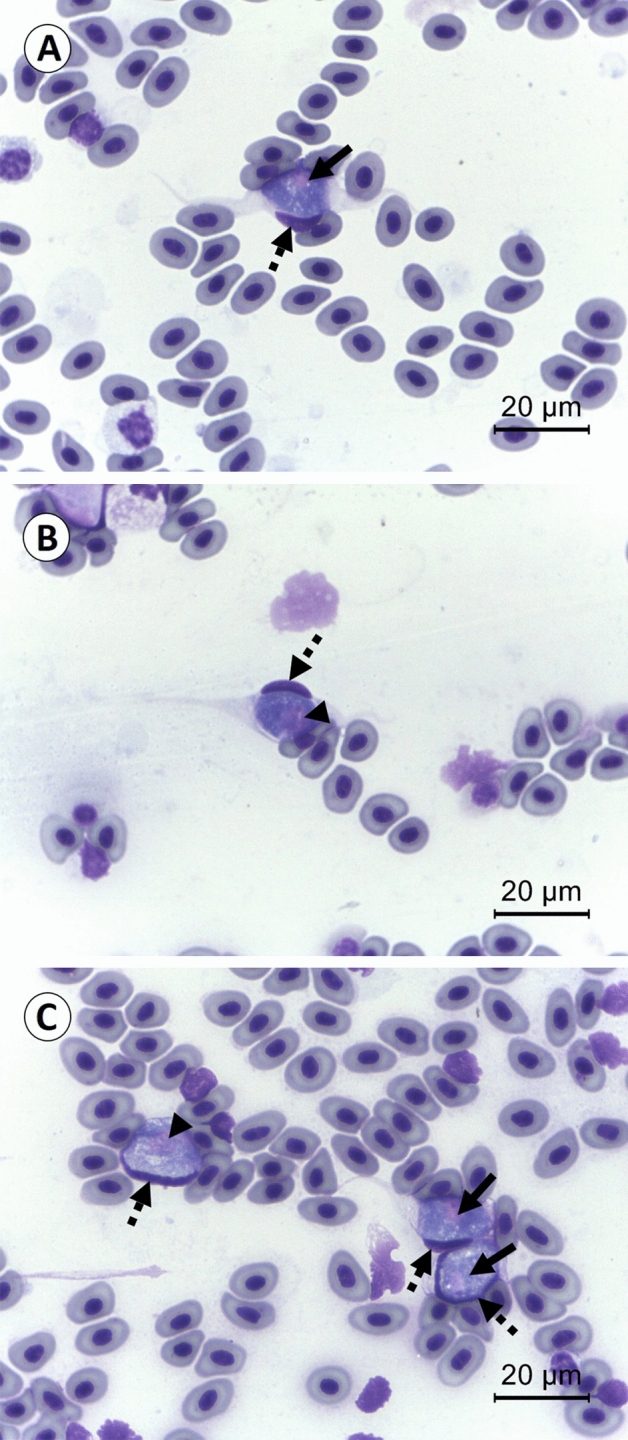
Gametocytes of *L. sabrazesi* in chicken’s cells identified from Geimsa stained blood smear. The high magnification micrographs showed the elongated macrogametocytes (**A**), elongated microgametocytes (**B**) and rounded microgametocyte (the left side) and rounded and elongated macrogametocytes (the right side) (**C**). The arrows present parasites’ nuclei of macrogametocytes. The arrowheads indicate parasites’ nuclei of microgametocytes. The dashed arrows demonstrate hosts’ nuclei.

Of the 313 chicken blood samples were examined under light microscope to observe the intra-erythrocytic *L. sabrazesi* gametocytes in blood smears (Fig. 1). Two hundred and fifty-two blood samples out of 313 (80.51%) were positive as investigated by the PCR analysis targeting *cytb* gene of *L. sabrazesi* (Table 1). The representative PCR products of parasite examined were 248 bp. The highest occurrence of *L. sabrazesi* infection (116/127; 91.34%) was found in Chiang Rai province, followed by Kanchanaburi province (72/86; 83.72%) and Phatthalung province (64/100; 64%) as shown in Table 1. The association between the occurence of *L. sabrazesi* infection and the factors, i.e., age (4, 4–12 and > 12 months), management system (backyard in households, free-range farms and non-evaporative cooling houses) and water sources (pond in the farm and river) nearby were considered to be statistically significant (*p* < 0.05), while the significant different was not found counting upon the chicken’s breed (native and layer), gender (male and female), type of feeds (commercial and natural) and insect control system (Table 2).

**Table 1:** Summary of *L. sabrazesi* infection in chickens from Chiang Rai, Kanchanaburi and Phatthalung provinces analyzed by PCR assay.

Parameters	Number of examined samples	Number of positive samples	Total (%)
**Chiang Rai province**	127	116	91.34
Mae Suai district			
**Kanchanaburi province**	86	72	83.72
Sai Yok district			
**Phatthalung province**	100	64	64.00
Mueang Phatthalung district	20	8	40.00
Srinagarindra district	16	13	81.25
Khuan Khanun district	42	31	73.81
Kong Ra district	22	12	54.55
Total	313	252	80.51

**Table 2:** Factors associated with *L. sabrazesi* infection of chickens in Chiang Rai, Kanchanaburi and Phatthalung provinces.

Parameters	Number of examined samples	Number of positive samples	Total (%)	χ^2^ (df)	*p*-value
**Breed**				2.7132 (1)	0 .099518
Native	150	115	76.67		
Layer	163	137	84.05		
Total	313	252	80.51		
**Age**				53.9465 (1)	< 0.00001
0–4 months	40	38	95.00		
4–12 months	182	164	90.11		
> 12 months	91	50	54.95		
Total	313	252	80.51		
**Gender**				0.0383 (1)	0.844754
Male	78	61	78.21		
Female	235	191	81.28		
Total	313	252	80.51		
**Management system**				31.0085 (2)	< 0.00001
Backyard in households	186	138	74.19		
Free-range farms	91	86	94.51		
Non-evaporative cooling houses	36	28	77.78		
Total	313	252	80.51		
**Type of feeds**				0.6267 (2)	0.731003
Commercial	36	28	77.78		
Natural	156	124	79.49		
Mixed	121	100	82.64		
Total	313	252	80.51		
**Water source**				18.4521 (1)	0.000017
Yes	255	217	85.10		
No	58	35	60.34		
Total	313	252	80.51		
**Insect control system**				1.2544 (1)	0.262717
Yes	25	18	72.00		
No	288	234	81.25		
Total	313	252	80.51		

χ^2^ = Chi-square; df = degree of freedom.

### Phylogenetic and similarity analysis of *L. sabrazesi cytb* gene sequences

**Figure 2:**
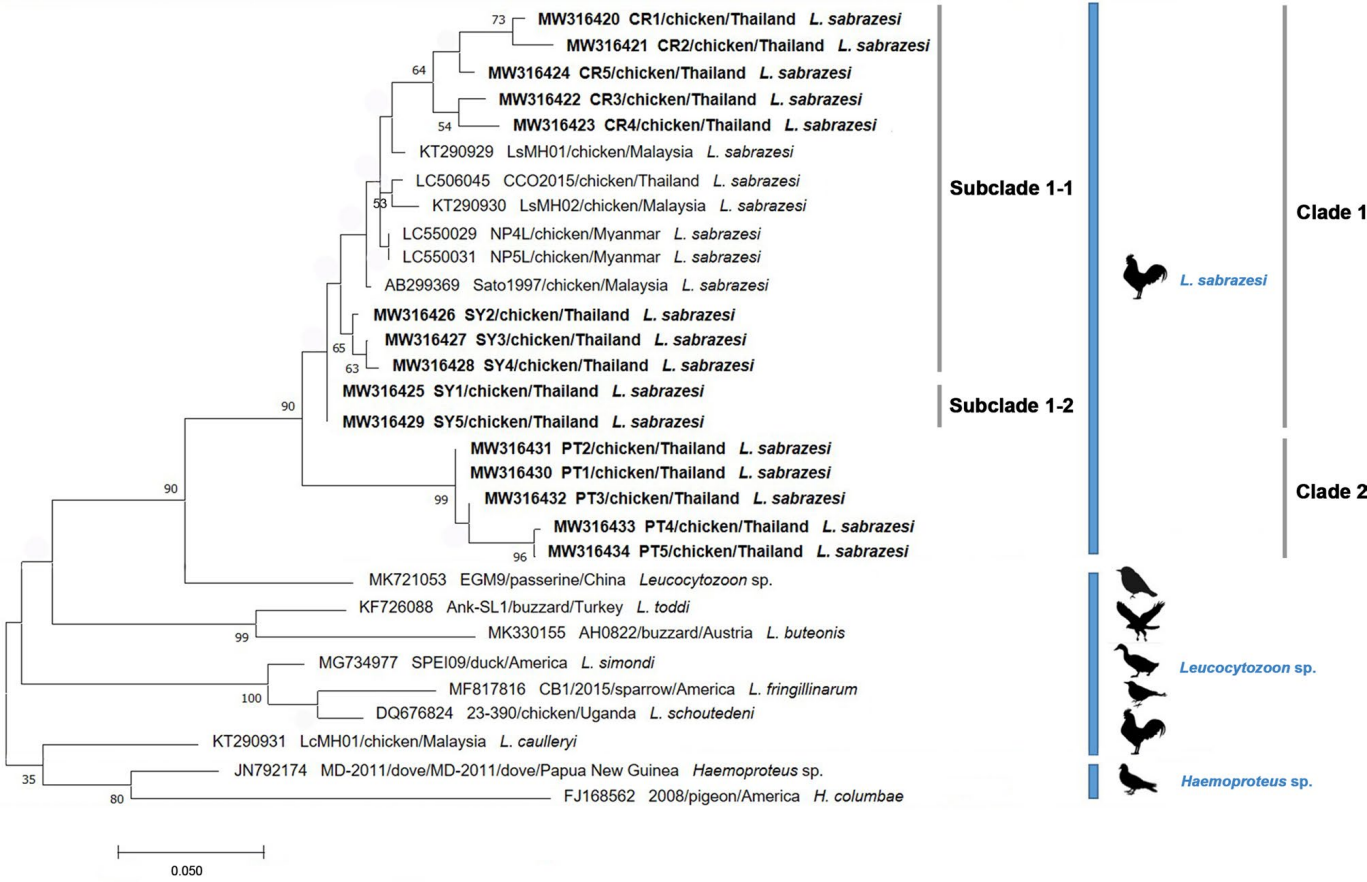
A Maximum Likelihood (ML) phylogenetic tree of *L. sabrazesi cytb* gene sequences in this study (blodface) and those taken from GenBank. The numbers on each node correspond to the bootstrap analysis of 1000 replicates (percentage more than 50% were listed). The GenBank assession numbers, the country and the parasite species name of the sequences are exhibited. Two gene sequences of *Haemoproteus* sp. were employed as the outer groups. The scale bar exhibits the number of substitutions per site.

In this study, the phylogenetic tree based on the alignment of the 15 Thailand sequences of *L. sabrazesi cytb* gene with 6 other sequences taken from the GenBank were classified as 2 clades. Clade 1 was divided into two subclades (subclade 1–1 and subclade 1–2). The sequences assigned to subclade 1–1 exhibited the genetic variability of *L. sabrazesi cytb* gene sequences from Chiang Rai and Kanchanaburi provinces of Thailand together with sequences from Malaysia, Thailand and Myanmar obtained from the GenBank. Subclade 1–2 was composed of two sequences from Kanchanaburi province and showed phylogenetic proximity. Five sequences from Phatthalung province of Thailand comprised clade 2 and also showed phylogenetic proximity. The *cytb* gene sequences among *L. sabrazesi* were highly conserved when compared with other strains as outer groups (Fig. 2). In addition, the similarity ranged between 89.5 and 100% for Thailand *cytb* sequences. The similarity of subclade 1–1 and subclade 1–2 was in the range of 93.3–99.5% and 94.3—100% among the Thailand *L. sabrazesi* sequences, respectively, while the similarity of the sequences within 2nd clade ranged between 89.5 to 100% (Table 3). The nucleic acid substitution rate in *cytb* sequences among *L. sabrazesi* was estimated under the Tamura and Nei^23^ mode as shown in Table 4.

**Table 3:** Similarity of the *L. sabrazesi cytb* gene sequences as detected in chicken sampled in Thailand.

Clade		Clade 1										Clade 2				
Subclade		Subclade 1–1								Subclade 1–2						
Acc. no		1	2	3	4	5	6	7	8	9	10	11	12	13	14	15
MW316420	1	100														
MW316421	2	98.1	100													
MW316424	3	97.2	96.3	100												
MW316422	4	96.8	95.8	96.7	100											
MW316423	5	95.3	96.3	96.2	97.7	100										
MW316426	6	94.3	94.8	95.8	95.3	95.3	100									
MW316427	7	93.8	94.3	95.3	94.8	94.8	99.5	100								
MW316428	8	93.3	94.3	94.8	94.3	94.7	99.1	99.5	100							
MW316425	9	94.3	94.3	95.8	96.2	94.7	99.1	98.6	98.2	100						
MW316429	10	94.3	94.3	95.8	96.2	94.7	99.1	98.6	98.2	100	100					
MW316431	11	92.8	92.3	91.2	92.3	90.8	92.8	92.3	91.8	93.8	93.8	100				
MW316430	12	92.8	92.3	91.2	92.3	90.8	92.8	92.3	91.8	93.8	93.8	100	100			
MW316432	13	92.3	91.8	90.7	91.8	90.2	92.3	92.8	92.4	93.3	93.3	99.5	99.5	100		
MW316433	14	90.2	90.2	89.5	90.7	89.7	91.3	91.8	91.8	92.3	92.3	96.7	96.7	97.2	100	
MW316434	15	90.2	90.2	89.5	90.7	89.7	91.3	91.8	91.8	92.3	92.3	97.2	97.2	97.7	99.5	100
Similarity (%)

**Table 4:** The nucleic acid substitution rate in *L. sabrazesi cytb* sequences as detected in chickens in Thailand and other countries.

Nucleic acid	A	T/U	C	G
**Sequence within Thailand**				
A	–	*4.27*	*1.80*	**10.57**
T/U	*3.35*	–	**12.13**	*1.30*
C	*3.35*	**28.70**	–	*1.30*
G	**27.15**	*4.27*	*1.80*	–
**Sequence worldwide**				
A	–	*3.96*	*1.68*	**11.41**
T/U	*3.15*	–	**11.49**	*1.19*
C	*3.15*	**27.04**	–	*1.19*
G	**30.10**	*3.96*	*1.68*	–

Each entry is the probability of substitution from one base (row) to another base (column). Rates of different transitional substitutions are shown in bold and those of transversional substitutions are shown in italics. The maximum Log likelihood for this computation was − 554.346 and − 588.782 for the sequences within Thailand and worldwide, respectively.

### Haplotype diversity

**Figure 3:**
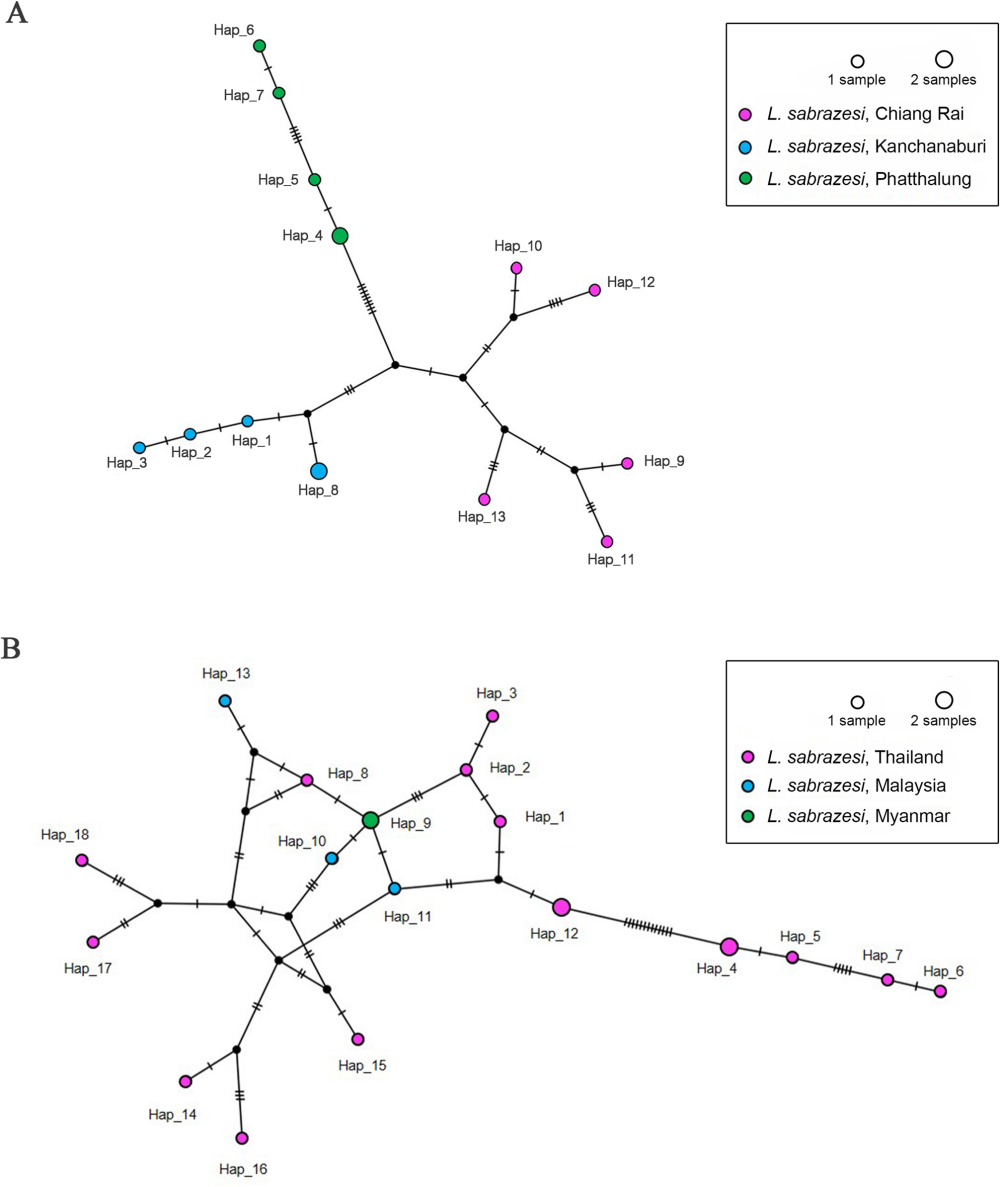
TCS network of haplotypes based on the *L. sabrazesi cytb* gene sequences detected in Thailand (**A**) and other countries (**B**).

The TCS Network tool was employed to construct the haplotype network of *L. sabrazesi cytb* gene sequences. Haplotype diversity based on *cytb* gene found in Chiang Rai, Kanchanaburi and Phatthalung provinces of Thailand was diverse when compared to worldwide gene sequences (Fig. 3). In Thailand, a total of 13 different haplotypes were analyzed including the 5 sequences (haplotype #1 to #3 and #8), 5 sequences (haplotype #4 to #7) and 5 sequences (haplotype #9 to #13) taken from chickens in Kanchanaburi, Phatthalung and Chiang Rai provinces, respectively. Bearing in mind, haplotype #4 was composed of 2 identical sequences (PT1 and PT2) and haplotype #8 was also comprised of SY1 and SY5 sequences (Fig. 3A and Table 5). In addition, 18 haplotypes indicated in TCS network exhibited that haplotypes #1 to #8, #12, #14 to #18 were detected in chicken in three provinces of Thailand. The rest of haplotypes was detected in other countries worldwide (Fig. 3B and Table 5).

**Table 5:** Polymorphism and genetic diversity of *L. sabrazesi cytb* gene sequences as detected in chickens in Thailand and other countries.

Gene	Size (bp)	*N*	VS	GC%	h	Dh (mean ± SD)	π (mean ± SD)	*K*
**Sequence within Thailand**								
*cytb*	222	15	26	29	13	0.981 ± 0.031	0.05393 ± 0.00428	11.9714
**Sequence worldwide**								
*cytb*	222	21	26	28.8	18	0.986 ± 0.019	0.04713 ± 0.00522	10.4619

*N:* number of analyzed sequences; VS: number of variable sites; GC: G × C content; h: number of haplotypes; Dh: diversity of haplotypes; SD: standard deviation; π: nucleotide diversity (per site); *K*: average number of nucleotide differences.

### Entropy analysis

**Figure 4:**
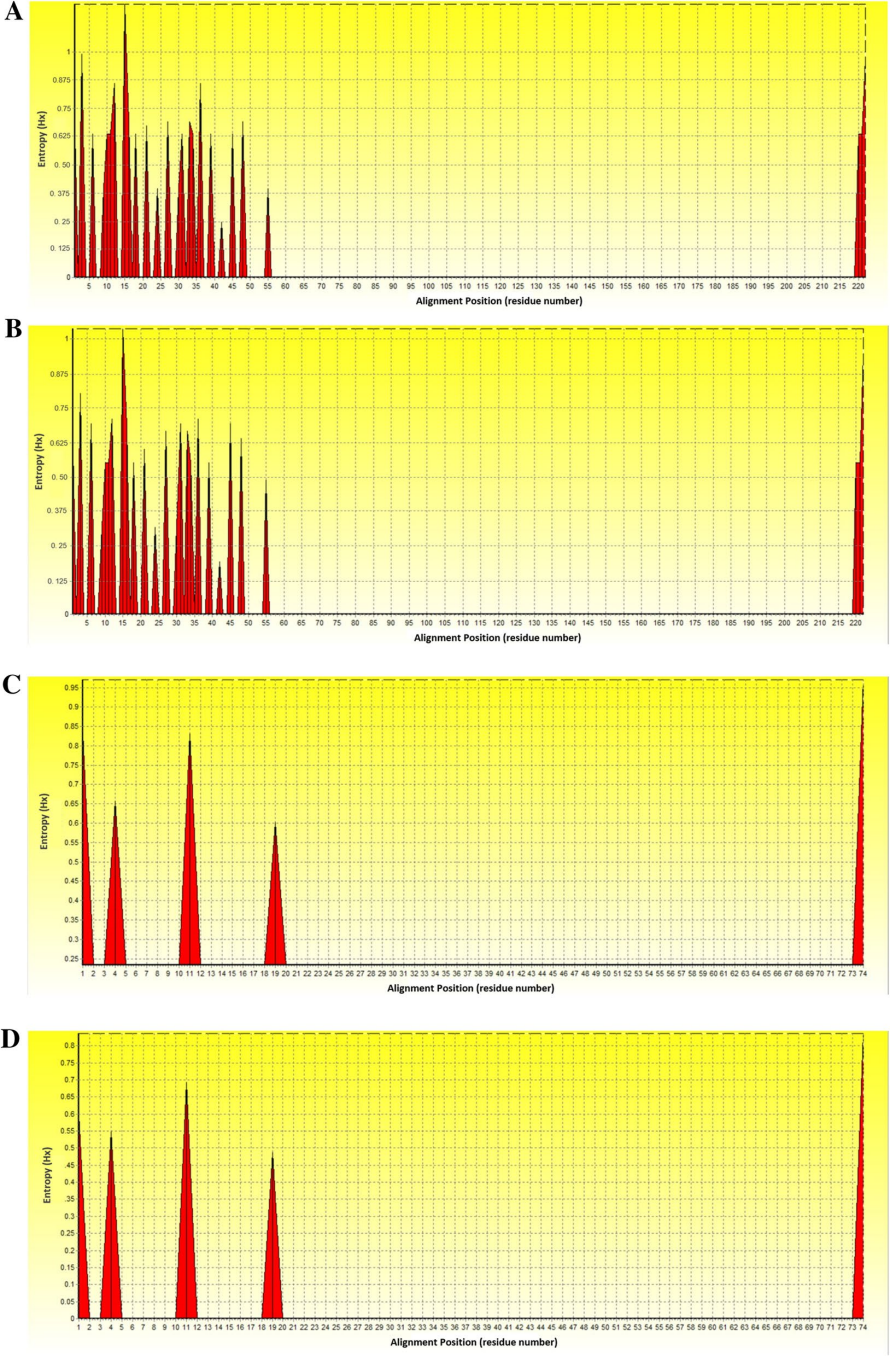
Nucleic and amino acid entropy plots obtained from *L. sabrazesi cytb* gene sequences. Entropy plot of nucleic acid sequences from Thailand (**A**) as well as Thailand and other countries (**B**). Entropy plot of amino acid sequences from Thailand (**C**) as well as Thailand and other countries (**D**).

Nucleic acid entropy analysis of 15 *L. sabrazesi cytb* gene sequences obtained in this study showed 26 high entropy peaks at nucleic acid alignment position 1, 3, 6, 9–12, 15–16, 18, 21, 24, 27, 30–31, 33–34, 36, 39, 42, 45, 48, 55 and 220–222 with entropy values ranged between 0.24493 and 1.21056 (Fig. 4A). The entropy plot of our 15 Thailand sequences aligned with 6 other sequences obtained from the GenBank exhibited the same nucleic acid alignment position but entropy values (0.19144–1.03570) were different (Fig. 4B). In addition, amino acid entropy analysis was performed using 15 Thailand *cytb* sequence alignments. The chart showed 5 high entropy peaks at amino acid alignment position 1, 4, 11, 19 and 74 with entropy values ranged between 0.39267 and 0.97012 (Fig. 4C). The entropy chart of 15 Thailand sequences with 6 other sequences taken from the GenBank showed the same amino acid alignment position, but entropy value (0.39267–0.97012) were different (Fig. 4D).

## Discussion

Leucocytozoonosis is an important disease causing clinical infection in chickens in many areas of the world, based on widespread distribution of the vector simuliid flies or culicoides midges. There are many conventional methods for detection of *Leucocytozoon* infection in chickens, such as clinical signs, hematological findings, microscopic examination of mature gametocytes in blood smears and serological tests. However, these methods require expertise since parasites are often missed when parasitemia are significantly low. Accurate diagnosis is needed to develop for appropriate treatment, transmission control and disease management. Thus, molecular assay, i.e., nested PCR, is a sensitive diagnostic method and used for detection and characterization of parasite DNA strains after gametocytes disappeared in host’s blood circulation.

Up to now, the detection of *Leucocytozoon* infection in chickens has only been reported in some areas of Thailand^18,19^. In addition, there is an obvious lack of relevant information on leucocytozoonosis isolated in chickens in Thailand. Our study is the first investigation that demonstrated a molecular occurrence of *L. sabrazesi* infection in chickens throughout Chiang Rai, Kanchanaburi and Phattalung provinces of Thailand.

In this study, our findings revealed a high molecular occurrence of *L. sabrazesi* in chicken blood samples in three regions (northern, western and southern) of Thailand. The molecular detection exhibited that of the animal sampled, 80.51% (252/313) were positive for *L. sabrazesi* based on the *cytb* gene (Table 1). In our work, the *cytb* gene has been used as a good marker for providing sufficient variation to establish phylogeographic patterns on the large scale and useful for epidemiological approaches of leucocytozoonosis^24,25^. The occurrence of *L. sabrazesi* infections was highest in chickens aged < 4 months (95%) compared to chickens aged 4–12 months (90.11%) and > 12 months (54.95%), which is in line with the previous studies by Naqvi et al.^26^, who showed the highest prevalence in chickens aged less than 6 months (24 weeks) was 78.8%. From our findings, it is possible that the intensive infection could be consistent with free-range farm system showing the higher statistically significant infection than other systems. Likewise, the climate condition, such as rainfall, are often involved with the occurrence of *L. sabrazesi* infection due to the longer survival of the parasite vectors (black flies and biting midges)^27^. Moreover, western and southern regions of Thailand have a long rainy season (about 3–4 months); this tropical area is a significant influence on the risk of *L. sabrazesi* infection and also seems to be a predisposing factor for the development of the parasite vectors which can survive in humid environment^9,26,28–30^.

Although the genetic diversity of *Leucocytozoon* sp. based on the sequences of *cytb* gene has been investigated in several countries^8,12,14,16,22,25,31^, little is known about the genetic diversity and the phylogeny of *L. sabrazesi* Thailand strain. In this study, the *cytb* gene in chicken population sampled in the northern, western and southern areas of Thailand was employed to determine the genetic diversity of *L. sabrazesi* in these regions. The phylogenetic analysis of chicken *L. sabrazesi cytb* gene Thailand isolate showed two clades together with the sequences from Malaysia and Myanmar. Our results exhibited that the genetic diversity observed in a phylogram was confirmed by the high similarity value for *L. sabrazesi cytb* gene (89.5–100%). This finding indicated high conserved sequences and phylogenetic proximity of *cytb* gene circulating in both Thailand and other countries.

In this work, the *cytb* gene sequence in blood samples of chickens in the northern, western and southern parts of Thailand were analyzed. Our results showed that the *L. sabrazesi* population was low diverse in Thailand, with the presence of probably more than one haplotype. The genotype of this gene was identified in haplotype networks. It was carried out with sequences detected in this study together with other sequences obtained from GenBank database that found in Malaysia and Myanmar. In this study, there has been mild different of morphological traits when compare the sequences from Chiang Rai haplotype to other haplotypes. Because Chiang Rai sequence has more prevalence and some chickens show pale comb or skinny correlate to amount of gametocytes in the blood smear. The severity of high prevalence is probably associated with areas where parasite’s vectors are abundant. The more prevalent haplotype is haplotype#12, which is 0–4 month age chickens. This indicated that there was some genetic diversity of *cytb* gene observed in the different haplotype networks in Thailand and other countries. Furthermore, the *cytb* sequence shared genetic traits with all sequences as ascertained previously from Malaysia and Myanmar. This finding exhibited that the genetic diversity among *L. sabrazesi* populations varied in accordance with the geographical area.

Regarding the analysis of *L. sabrazesi* nucleic acid sequences, our results approved the polymorphism with 26 entropy peaks reaching up to 1.21 (sequence within Thailand) and 1.04 (sequence worldwide). In addition, our results showed 5 entropy peaks of amino acid sequences reaching up to 0.97 (sequence within Thailand) and 0.84 (sequence worldwide). These indicated that different genotypes may involve being a genetic diversity of *L. sabrazesi* distribution in Thailand.

## Conclusions

This study is the first report indicating a molecular occurrence and genetic diversity of *L. sabrazesi* in chicken blood samples in Thailand. Our findings showed that *L. sabrazesi cytb* gene is genetically conserved in Thailand and other countries. These could help to ameliorate the understanding of phylogeny and genetic diversity among *cytb* gene of *L. sabrazesi* Thailand strain. Therefore, the periodical assessment of the occurrence of leucocytozoonosis is necessitate to control the affectivity of the effective treatment and prevention throughout the country to reduce the infection of chicken vector-borne parasites.

## Methods

### Study locations and sample sizes

**Figure 5:**
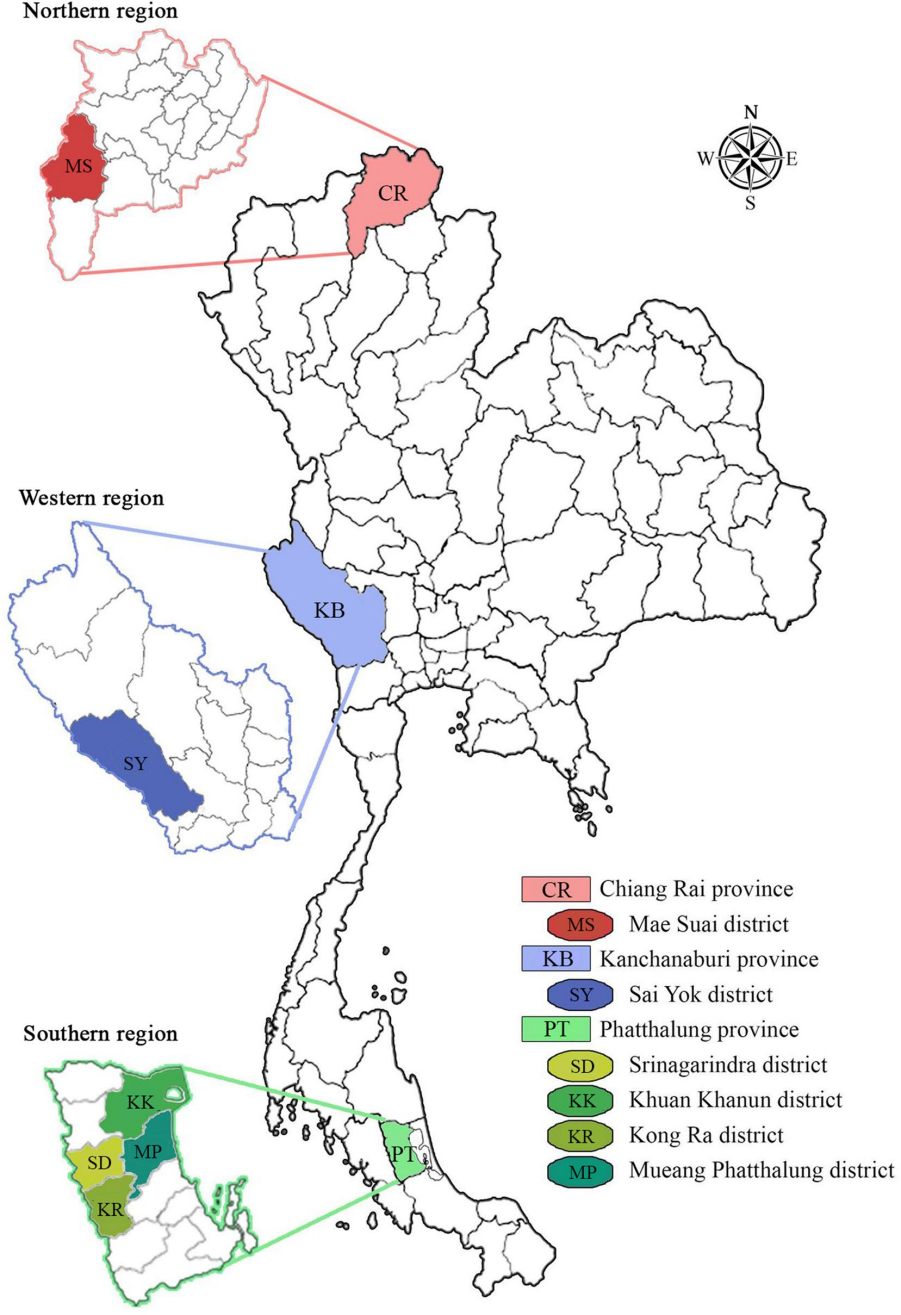
Geographical location of northern, western and southern regions where chicken blood samples are collected and examined. Legends indicate the distribution of *Leucocytozoon sabrazesi* Thailand strains discriminated in chickens from Mae Suai district (MS) in Chiang Rai province (CR), Sai Yok district (SY) in Kanchanaburi province (KB) and Srinagarindra (SD), Khuan Khanun (KK), Kong Ra (KR) and Mueang Phatthalung (MP) districts in Phatthalung province (PL).

The present study was conducted in three regions of Thailand (Fig. 5). Chicken blood samples were collected in Mae Suai district (19°39′24″N, 99°32′30″E) of Chiang Rai province in northern region, in Sai Yok district (14°6′56″N, 99°8′40″E) of Kanchanaburi province in western region, and in four districts including Srinagarindra (7°34′24″N, 99°56′30″E), Khuan Khanun (7°44′6″N, 100°0′36″E), Kong Ra (7°24′12″N, 99°57′0″E) and Mueang Phatthalung (7°37′6″N, 100°4′24″E) of Phatthalung province in southern region. Total 313 blood samples were randomly collected from the chickens raised in both backyard in households, free-range farms and non-evaporative cooling houses during December 2019 and October 2020. The sample sizes were calculated using the formula based on an equation, *n* = *t*^2^ × *p*(1 *−* *p*)*/m*^2^, inserting the following values: the prevalence of *L. sabrazesi* infection among chickens in Thailand (*p*)^18,19^, a 95% confidence level (t) and 5% margin of error (*m*).

### Collection of blood samples and microscopic examination

Between 0.3 and 2 ml of blood was collected via either the brachial wing vein or medial metatarsal vein of each chicken. They were kept into the centrifuge tube containing lithium heparin to preclude coagulation, and stored at − 80 °C until further used. In addition, some thin fresh blood smears were prepared for each chicken on glass slides, air-dried, fixed in 100% methanol for 1 min, and then stained with Giemsa stain (SIGMA-ALDISH, Germany) for 40 min.

### DNA extraction

Genomic DNA of *L. sabrazesi* was extracted from all collected blood samples of chickens using Tissue DNA Extraction Kit (OMEGA, bio-tek, USA) following the protocol of Watthanadirek et al.^32^ and Junsiri et al.^33^ with some modifications. Briefly, 20 µl of blood samples were mixed with 25 µl of OB Protease solution and 250 µl of BL buffer and incubated at 70 °C for 1 min. After adding with 250 µl of 100% ethanol, samples were transferred to HIBIND DNA Mini Column. Finally, DNA samples were eluted in 50 µl Milli-Q water and kept at − 20 °C until used. The concentration and purity of DNA were defined with NANODROP 2000 Spectrophotometers (THERMO SCIENTIFIC) at 260/280 and 260/230 ratios.

### Amplification and detection of *L. sabrazesi* DNA

The specific primer pairs designed from *L. sabrazesi cytb* sequence submitted in GenBank database under accession numbers AB299369.1, were utilized to amplify DNA fragments of the *cytb* gene. *L. sabrazesi cytb* gene was amplified by nested PCR using 2 pairs of specific primers. In the first step of amplification, the primers, namely LsF1 (5′-CATATATTAAGAGAATTATGGAG-3′) and LsR1 (5′-ATAAAATGYTAAGAAATACCATTC-3′) were used. In the second step, the primers, namely LsF2 (5′-TAATCACATGGGTTTGTGGA-3′) and LsR2 (5′-GCTTTGGGCTAAGAATAATACC-3′) were also used. The expected size of amplification products was 248 bp.

PCR reaction mixtures consisting of 50 ng DNA template, 0.2 µM each of the primers, 0.125 mM of each deoxynucleoside triphosphate (dNTPs), 3 mM MgCl_2_, 0.25 U *Tag* DNA polymerase (NEW ENGLAND BIOLABS, UK), 1× standard Tag reaction buffer and nuclease free water, were put through in a thermal cycle (BIO-RAD, USA) with the following condition: 40 cycles of denaturation at 94 °C for 1 min (1st step) and for 20 s (2nd step), annealing at 50 °C for 1 min (1st step) and 53 °C for 20 s (2nd step), extension at 68 °C for 1 min (1st step) and for 30 s (2nd step) as well as a final extension at 68 °C for 5 min (1st and 2nd steps). PCR product was observed by 1.2% agarose gel stained with Fluorostain DNA Fluorescent Staining Dye (SMOBIO, Taiwan) and viewed under ultraviolet (UV) transilluminator. A 100 bp Plus DNA Ladder (THERMO FISHER SCIENTIFIC, USA) was used as standard for defining the molecular mass of PCR products.

### Cloning of the mitochondrial *cytb* gene from *L. sabrazesi* DNA

*L. sabrazesi cytb* gene was cloned into vector with following specific primers: LsF 5′-CACCTAATCACATGGGTTTGTGGA-3′ and LsR 5′-GCTTTGGGCTAAGAATAATACC-3′. The 4 nucleotides (CACC) were added at 5′ end of forward primer with the overhang sequence (GTGG) in pET100/D-TOPO^®^ vector (INVITROGEN, USA) to enable directional cloning. The PCR reaction was conducted with the protocols as described in previous section. PCR products were purified using ULTRACLEAN 15 DNA Purification Kit (MO BIO LABORATORIES, USA) following the manufacturer’s instructions for cloning. The 20 ng of the blunt-end PCR products were inserted in the pET100/D-TOPO vector (INVITROGEN LIFE TECHNOLOGIES, USA). Then 3 µl of the cloning reactions were transformed into chemically competent *Escherichia coli* cells (INVITROGEN, USA). Subsequently, 200 µl of transformed bacterial culture was spread on the Luria Bertani (LB) agar plates containing 100 µg ampicillin and incubated for overnight at 37 ºC. The positive clones were selected and grown in LB medium containing ampicillin for overnight. Finally, the recombinant plasmids (pET100-*cytb*) were extracted from the competent cells using AXYPREP Plasmid Miniprep Kit (AXYGEN BIOSCIENCE, USA) following the manufacturer’s instructions and analyzed for correctly sized inserts by agarose gel electrophoresis^32,33^.

### Sequencing analysis

Purified PCR products were confirmed by Sanger method of DNA sequencing. All DNA sequences were analyzed by BLAST (The National Center for Biotechnology Information, NCBI, http://www.ncbi.nlm.nih.gov/BLAST). All sequences were deposited in GenBank, accession numbers are provided in Table 6.

**Table 6:** The *L. sabrazesi cytb* nucleotide sequences amplified in Thailand strains were deposited in GenBank database.

Regions	Provinces	Districts	Animal ID	GenBank accession numbers
Northern	Chiang Rai	Mae Suai	CR1	MW316420
			CR2	MW316421
			CR3	MW316422
			CR4	MW316423
			CR5	MW316424
Western	Kanchanaburi	Sai Yok	SY1	MW316425
			SY2	MW316426
			SY3	MW316427
			SY4	MW316428
			SY5	MW316429
Southern	Phatthalung	Khuan Khanun	PT1	MW316430
			PT2	MW316431
		Kong Ra	PT3	MW316432
		Mueang Phatthalung	PT4	MW316433
		Srinagarindra	PT5	MW316434

### Phylogenetic sequence analysis

*Leucocytozoon sabrazesi cytb* gene sequences were used for sequence alignment and phylogenetic analysis as shown in Table 6 and two gene sequences of *Haemoproteus* sp. were employed as the outer groups (accession no. JN792174 and FJ168562). Multiple sequence alignment was proceeded with Clustal W algorithm and then genetic inference was carried out by Maximum Likelihood (ML) phylogenetic tree using MEGA software version 7.0.2.6^34,35^. Bootstrap analysis with 1000 repetitions was employed to evaluate the confidence of the branching pattern of the trees^36^. The distance analyses were performed using the Kimura 2-parameter distance model^37^. The similarity was determined with the pairwise-distance method^38^.

### Haplotype diversity

The obtained alignment of *L. sabrazesi cytb* gene sequences was used to estimate the nucleotide diversity (π), diversity of haplotypes (Dh), number of haplotypes, and the average number of nucleotide differences (*K*), using the DnaSP version 6.0 software^39^. Likewise, the nucleotide sequences were put through to the Population Analysis with the Reticulate Trees (popART) program^40^ in order to analyze the TCS Network construction^41^.

### Entropy analysis

Entropy analysis was employed to verify the variability of the nucleic acid and amino acid sequences. *L. sabrazesi cytb* nucleotide sequences were translated into amino acids, aligned and then analyzed by the Entropy (H (x)) plot using Bioedit version 7.2.6.1^42^.

### Statistical analysis

The overall occurrence was calculated as a percent of number of *L. sabrazesi*-infected animals in the total number of examined animals. Relative occurrence of this parasite was calculated and proceeded by breed, age, gender, management system, type of feed, water source and insect control system. For all analyses, confidence level was obtained at 95% and *p*-value of ≤ 0.05 was considered to be the level of significance. Statistical analysis was defined using SPSS software for Window version 22.0 (SPSS Inc., Chicago, USA).

### Ethic approval and permit

All experimental procedures regarding animals were carried out under the following approval and permit from the Animal Care and Use Committee (IMBMU-ACUC), Institute of Molecular Biosciences, Mahidol University, Thailand. All methods were performed in accordance with relevant guidelines and regulations.
