# Author Correction: Molecular detection and genetic diversity of *Leucocytozoon sabrazesi* in chickens in Thailand

**DOI:** 10.1038/s41598-024-53903-6

**Published:** 2024-02-09

**Authors:** Pacharaporn Khumpim, Runglawan Chawengkirttikul, Witchuta Junsiri, Amaya Watthanadirek, Napassorn Poolsawat, Sutthida Minsakorn, Nitipon Srionrod, Panat Anuracpreeda

**Affiliations:** 1 Parasitology Research Laboratory (PRL), Institute of Molecular Biosciences, Mahidol University, Nakhon Pathom, 73170 Thailand; 2 Department of Microbiology, Faculty of Science, Mahidol University, Bangkok, 10400 Thailand

Correction to: *Scientific Reports* 10.1038/s41598-021-96241-7, published online 17 August 2021

The original article contained errors. Pacharaporn Khumpim was omitted from the author list in the original version of this Article.

The acknowledgement now reads:

This research project is financially supported by Mahidol University (Basic Research Fund: fiscal year 2021) and Research Grants from the National Research Council of Thailand (NRCT) and the Mid-Career Research Grant co-funded by National Research Council of Thailand (NRCT) and Mahidol University [Grant Number NRCT5-RSA63015-23] to Panat Anuracpreeda, and Royal Golden Jubilee Ph.D. (RGJ-PHD) Scholarship [Grant Number PHD/0050/2560] to Pacharaporn Khumpim.

The Author Contributions section now reads:

“P.K. was involved with all aspects of the planning and conducting of this research including the laboratory work, processing of data and drafting of manuscript. R.C., W.J., A.W., N.P., S.M. and N.S. contributed to study design, conducting of the laboratory work and manuscript revision. P.A. was involved in all aspects of the project from research design, laboratory work, data analysis, laboratory resources, funding acquisition, editing of manuscript, manuscript development and final revisions. All authors reviewed the manuscript.”

The original Article has been corrected.

